# Performance of liver stiffness measurements obtained with FibroScan is affected by glucose metabolism in patients with nonalcoholic fatty liver disease

**DOI:** 10.1186/s12944-021-01453-5

**Published:** 2021-03-23

**Authors:** Xinyu Yang, Xinxia Chang, Shengdi Wu, Xiaoyang Sun, Xiaopeng Zhu, Liu Wang, Yushan Xu, Xiuzhong Yao, Shengxiang Rao, Xiqi Hu, Mingfeng Xia, Hua Bian, Hongmei Yan, Xin Gao

**Affiliations:** 1 Department of Endocrinology and Metabolism, Zhongshan Hospital, Fudan University, Shanghai, China; 2 Fudan Institute for Metabolic Disease, Fudan University, Shanghai, China; 3 Department of Gastroenterology, Zhongshan Hospital, Fudan University, Shanghai, China; 4 Department of Endocrinology and Metabolism, The First Affiliated Hospital of Kunming Medical University, Kunming, China; 5 Department of Radiology, Zhongshan Hospital, Fudan University, Shanghai, China; 6 Department of Pathology, Shanghai Medical College, Fudan University, Shanghai, China; 7 Department of Endocrinology and Metabolism, Wusong Branch of Zhongshan Hospital, Fudan University, Shanghai, China

**Keywords:** Nonalcoholic fatty liver disease, Abnormal glucose metabolism, FibroScan, Liver stiffness measurement, Fibrosis

## Abstract

**Background:**

The performance of liver stiffness measurements (LSMs) obtained using FibroScan can be affected by several factors, and cut-off values are different for fibrosis caused by various aetiologies. The study aims to evaluate the diagnostic accuracy of LSM in nonalcoholic fatty liver disease (NAFLD) patients with abnormal glucose metabolism and investigate whether the LSM value would be affected by metabolic indicators.

**Methods:**

The study involved 91 NAFLD patients with abnormal glucose metabolism who underwent liver biopsy. The diagnostic accuracy of LSM value was evaluated by the receiver operator characteristic (ROC) curves, with the biopsy results taken as the gold standard. Multivariate linear regression and subgroup analysis were performed to determine the correlated indicators.

**Results:**

The areas under the ROC curves (AUROCs) of LSM values for detecting fibrosis stage ≥1, 2, 3 and 4 were 0.793 (95% confidence interval [CI]: 0.695–0.871), 0.764 (95% CI: 0.663–0.846), 0.837 (95% CI: 0.744–0.906) and 0.902 (95% CI: 0.822–0.955), with cut-off values of 6.3, 7.6, 8.3 and 13.8 kPa, respectively. Multivariate linear regression demonstrated that haemoglobin A1c (HbA1c, β = 0.205, *P* = 0.026) and alanine aminotransferase (ALT, β = 0.192, *P* = 0.047) were independently associated with the LSM value after adjustment for fibrosis stage, ballooning and inflammation grade from liver biopsy. Subgroup analysis demonstrated that LSM values were slightly higher in patients with HbA1c ≥7% than in those with HbA1c < 7% and in patients with body mass index (BMI) ≥30 kg/m^2^ than in those with BMI < 30 kg/m^2^.

**Conclusions:**

FibroScan was valuable for the evaluation of liver fibrosis in NAFLD patients with abnormal glucose metabolism. FibroScan is recommended to evaluate severe fibrosis, especially to exclude advanced fibrosis. Glucose metabolism state may affect LSM values.

**Supplementary Information:**

The online version contains supplementary material available at 10.1186/s12944-021-01453-5.

## Introduction

Nonalcoholic fatty liver disease (NAFLD) has become the predominant cause of chronic liver injury worldwide and refers to the presence of ≥5% hepatic steatosis (HS) without other competing aetiologies, including chronic viral hepatitis, excessive alcohol consumption, the use of steatogenic medication or hereditary disorders [1]. Its spectrum ranges from nonalcoholic fatty liver (NAFL) and nonalcoholic steatohepatitis (NASH) to cirrhosis or even carcinoma, and it is associated with features of metabolic syndrome, such as hypertension, insulin resistance, diabetes mellitus (DM) and dyslipidaemia. NAFLD increases risks of cardiovascular disease and accelerates the progression of underlying disease, leading to severe consequences [2–4]. NAFLD patients with DM are prone to develop NASH, liver fibrosis and cirrhosis, and even liver cancer. The overall prevalence of NAFLD among patients with type 2 diabetes mellitus (T2DM) is 55%, more than 2-fold higher than that in the general population, with a very high rate of NASH [5–7]. In the course of NAFLD, liver fibrosis, an important predictor of adverse prognosis, is the most relevant target for early diagnosis and treatment, as it has been associated with further deterioration of cirrhosis and increased overall mortality [8, 9]. In the authors’ recent study, fibrosis occurred in up to 50% of patients with both NAFLD and T2DM [10]. Considering the large number of patients with T2DM, the burden of NAFLD management appears enormous. Although effective therapies for NAFLD have not been established, early intervention can significantly improve its poor prognosis. Therefore, the accurate and early detection of NAFLD in patients with abnormal glucose metabolism, especially the determination of fibrosis stage of liver fibrosis, is crucial [8, 11, 12].

Liver biopsy, an invasive procedure, has been recommended as the gold standard for the diagnosis and classification of NAFLD, but the limitations of possible bleeding risks and sampling errors make it unsuitable for screening and frequent monitoring [13–15]. Therefore, noninvasive alternatives to liver biopsy have been investigated, such as serum biomarkers, clinical scoring systems and imaging tests, including ultrasonography, proton magnetic resonance spectroscopy (^1^H-MRS), magnetic resonance imaging-derived proton density fat fraction (MRI-PDFF), FibroScan and magnetic resonance elastography (MRE) [16, 17]. Among them, FibroScan (transient elastography; EchoSens, Paris, France) is recommended by the *2018 NAFLD guidance* because of its convenience, clinical accessibility, low cost and simultaneous measurement of fibrosis and steatosis^16^. Liver stiffness measurement (LSM) obtained using FibroScan is one parameter for the diagnosis and quantification of liver fibrosis by measuring mechanical or ultrasound shear wave propagation through the hepatic parenchyma [18, 19].

Several studies have assessed the diagnostic performance of FibroScan, most of which targeted patients with chronic hepatitis B [20, 21], while others focused on NAFLD [18, 22, 23]. *Transient elastography expert consensus* pointed out that there are differences in the cut-off values in patients with liver fibrosis caused by various aetiologies, consisting of hepatitis B, hepatitis C and NAFLD. Factors such as liver inflammation activity manifested by alanine aminotransferase (ALT) or increased bilirubin levels, excessive alcohol intake and eating may lead to an increase in LSM values [24]. In view of the promoting effect of abnormal glucose metabolism on liver disease, NAFLD patients with abnormal glucose metabolism may have different specific cut-off values than general NAFLD patients. However, to date, there have been no studies focusing on these populations and the lack of cut-off values.

The study aims to evaluate the diagnostic performance of FibroScan and obtain cut-off values in NAFLD patients with abnormal glucose metabolism and to investigate whether metabolic indicators would affect the measurement of FibroScan to provide clinical advice for the application of FibroScan in the diagnosis and evaluation of NAFLD patients.

## Material and methods

### Design and subjects

This cross-sectional study included 91 NAFLD patients evaluated in Zhongshan Hospital, Fudan University, between July 2015 and December 2019, 89 of whom underwent liver biopsy, while 2 others had cirrhosis based on ultrasound. The time interval between FibroScan examination and biopsy was < 2 weeks. Patients with a history of excessive alcoholic consumption (> 20 g for men or > 10 g for women/day), chronic viral hepatitis, drug use and any other causes associated with liver injury were excluded. The entire process of the study was conducted according to the ethical guidelines of the Declaration of Helsinki and was approved by the Human Research Ethics Committee of Zhongshan Hospital Clinic (B2021-130). All patients have signed an informed consent before study.

### Liver biopsy and histopathologic evaluations

Liver biopsy samples were obtained using a 16-gauge needle from the right liver lobe of NAFLD patients under ultrasound guidance. Two specimens were obtained from each person to ensure a sample size sufficient for analysis and to reduce error. All biopsy specimens were evaluated by two experienced pathologists blinded to the clinical and biological data. Histopathological findings were reported in accordance with the guidelines of the Pathology Working Group of NASH Clinical Research Network of the National Institutes of Health [25]. The NAFLD activity score (NAS) consists of three parts: 1) hepatic steatosis grade 0–3, 2) lobular inflammation grade 0–3, 3) hepatocyte ballooning grade 0–2. Fibrosis was staged from 0 to 4. The classification criteria are described in Table 1. NASH was defined as NAS > 4 or NAS =4 with the manifestation of steatosis, inflammation, and ballooning at the same time [25, 26].

**Table 1:** Basic characteristics and hepatic histopathology of the patients

Characteristics	NAFLD
Total	91
Sex, Male/Female	46/45
Age, y	40 (32–56)
Weight, kg	81.55 ± 15.32
BMI, kg/m^2^	29.10 ± 4.06
Waist–hip ratio	0.94 ± 0.06
T2DM, %	65 (71.4%)
Hypertension, %	29 (31.9%)
Platelet, /10^5^ μL	238.34 ± 70.05
Fasting glucose, mmol/L	5.8 (5.1–7.0)
2 h glucose, mmol/L	12.47 ± 3.82
Haemoglobin A1c, %	6.7 (5.8–8.1)
Triglycerides, mmol/L	1.84 (1.23–2.68)
Total cholesterol, mmol/L	4.39 (3.89–5.14)
LDL cholesterol, mmol/L	2.57 ± 0.85
HDL cholesterol, mmol/L	0.99 (0.86–1.12)
Albumin, g/L	44 (42–47)
Alanine aminotransferase, U/L	61 (43–89)
Aspartate aminotransferase, U/L	37 (27–49)
C-reactive protein, mg/L	1.9 (1.1–3.7)
LSM, kPa	8.5 ± 3.5
Steatosis grade, n (%)	
1 5–33%	18 (20.2)
2 33–66%	52 (58.4)
3 > 66%	19 (21.4)
Lobular inflammation, n (%)	
0 None	3 (3.4)
1 < 2 foci per 200× field	35 (39.3)
2 2–4 foci per 200× field	38 (42.7)
3 > 4 foci per 200× field	13 (14.6)
Liver cell ballooning, n (%)	
0 None	2 (2.3)
1 Few balloon cells	14 (15.7)
2 Many balloon cells	73 (82.0)
NAFL/NASH, n	13/76
Fibrosis stage, n (%)	
0 None	8 (8.8)
1 Perisinusoidal or periportal	33 (36.2)
2 Perisinusoidal and portal/periportal	30 (33.0)
3 Bridging fibrosis	15 (16.5)
4 Cirrhosis	5 (2 diagnosed with ultrasound) (5.5)

All data are expressed as the mean ± SD, medians (interquartile range), or n (%), as appropriate

*Abbreviations: *BMI* body mass index, *LSM* liver stiffness measurement

### LSM measurement

LSM measurement was performed with FibroScan using the M probe, as in most previous studies [18, 23]. Details of measurement are described in several previous studies, performed with the same machine by the same experienced operator blinded to other noninvasive methods and biopsy results [23, 27]. The examination duration was less than five minutes. Ten valid measurements were obtained from each patient and then the success rate, the ratio of the successful measurement times over the total times, was calculated. The result was considered reliable only when the success rate was ≥60% and the interquartile range (IQR)/median was ≤30%. The median value was kept as a representative result. Liver stiffness measurement results are expressed in kilopascals (kPa).

### Basic characteristics collection

The medical history of each patient was collected, including general physical characteristics, history of chronic diseases and history of medication. Hypertension was diagnosed according to criteria [28]. Routine serological tests were performed upon admittance. Fasting blood samples were collected locally and then shipped to the clinical laboratory of Zhongshan Hospital for assessment of blood glucose, lipid profiles and other blood biochemical parameters. Abnormal glucose metabolism was defined as fasting glucose ≥5.6 mmol/L or 2 h glucose ≥7.8 mmol/L according to 2003 guidelines of the American Diabetes Association [29, 30].

### Statistical analysis

Continuous variables with a normal distribution are summarized as the mean ± SD, while those without a normal distribution are described as the median (interquartile range). Categorical variables were summarized as frequencies and percentages. SPSS software (version 23.0) was used for data analysis. The graphs were generated with GraphPad (version 8.4) and MedCalc (version 19.1). The diagnostic accuracy was evaluated by the receiver operator characteristic (ROC) curves. The area under the ROC curve (AUROC) and the cut-off value optimized with Youden’s index for each degree were calculated, with sensitivity, specificity, positive predictive value (PPV) and negative predictive value (NPV) obtained. The unpaired t test and Kruskal-Wallis test with Dunn’s multiple correction were used for univariate comparisons between groups. The independent correlation was analysed using multivariate linear regression analysis. Spearman’s rank correlation coefficient was used to assess the correlation between the liver histopathologic degree and FibroScan results in patients who underwent biopsy. A *P* value < 0.05 was considered statistically significant.

## Results

### Patient characteristics

In this study, a total of 91 patients with NAFLD were involved. All patients received blood biochemical examination. LSM and CAP values obtained using FibroScan were attempted in all patients. The physical, clinical, serological, and histologic characteristics are detailed in Table 1.

### Evaluation of diagnostic accuracy of FibroScan on liver fibrosis in patients with NAFLD

**Fig. 1:**
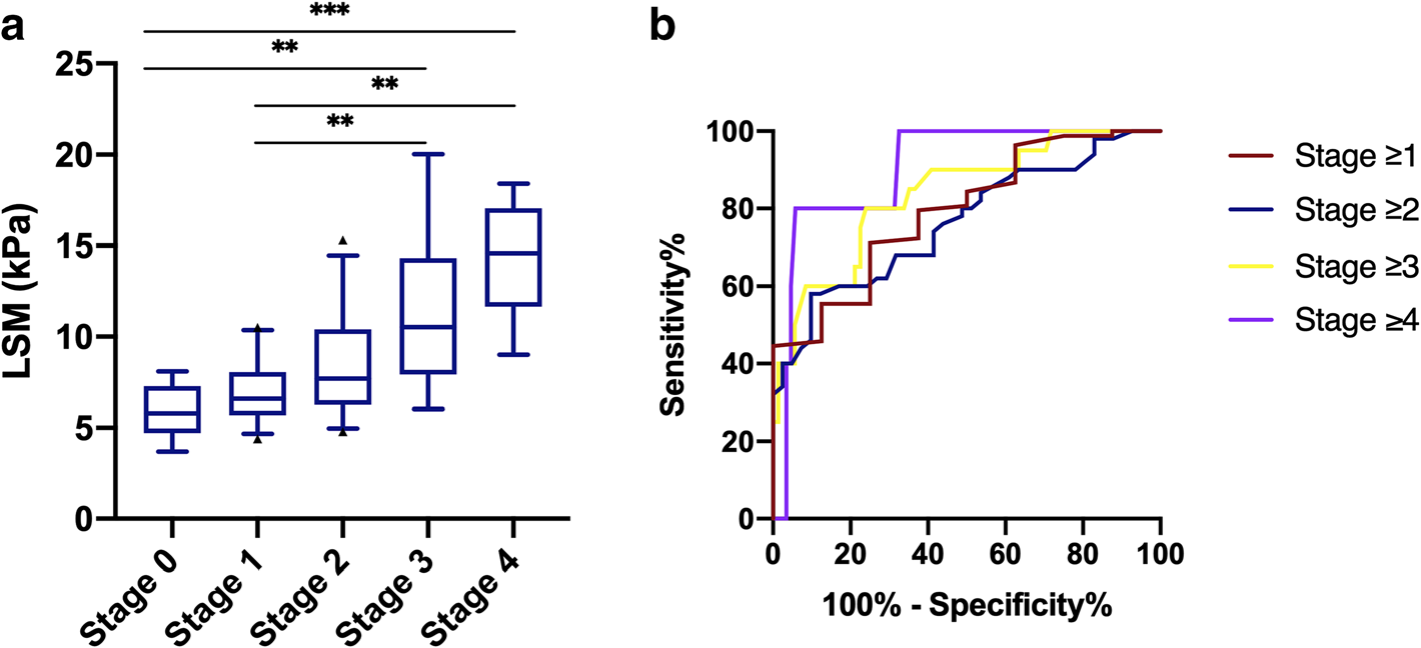
**a** The liver stiffness measurement (LSM) values were grouped by the liver fibrosis stage. The abscissa represents the stage of fibrosis, and the ordinate represents the LSM value. It reveals significant stepwise increases in the LSM with increasing histologic severity of hepatic fibrosis. Boxplots are shown as the median, interquartile range, and 5 and 95% percentiles. ▲ represents the value of greater variability. The Kruskal-Wallis test with Dunn’s multiple correction was used for univariate comparisons between groups. * represents *P* < 0.05, ** represents *P* < 0.01, *** represents *P* < 0.001. **b** The diagnostic performance of the LSM values for liver fibrosis stage. Receiver operator characteristic (ROC) curves were shown regarding the performance of LSM in distinguishing liver fibrosis stage 0 from 1 to 4, 0–1 from 2 to 4, 0–2 from 3 to 4, and 0–3 from 4

The LSM was used to assess the stage of liver fibrosis measured by FibroScan in patients with NAFLD. The median LSM values for stages 0, 1, 2, 3, and 4 were 6.15, 6.60, 7.70, 10.50 and 14.60 kPa, respectively. The results are shown in Fig. 1a and revealed significant stepwise increases in the LSM with increasing histologic severity of hepatic fibrosis. To investigate the diagnostic accuracy of FibroScan, the ROC curves were differentiated between liver fibrosis at stage 0 vs 1–4, 0–1 vs 2–4, 0–2 vs 3–4, and 0–3 vs 4, as shown in Fig. 1b. The AUROCs in diagnosing liver fibrosis stages 1, 2, 3 and 4 were 0.793 (95% confidence interval [CI]: 0.695–0.871), 0.764 (95% CI: 0.663–0.846), 0.837 (95% CI: 0.744–0.906) and 0.902 (95% CI: 0.822–0.955), respectively. Optimized with Youden’s index, the cut-off values of LSM were 6.3, 7.6, 8.3, and 13.8 kPa in detecting stage 1, 2, 3, and 4 liver fibrosis in NAFLD patients with abnormal glucose metabolism. The results are detailed in Table 2. It seemed that the diagnostic accuracy of LSM improved as the histologic severity of hepatic fibrosis increased. The diagnostic AUROC at stage 4 even reached up to 0.902, indicating that FibroScan is an ideal machine to evaluate the severity of liver fibrosis, especially to more severe degrees. In addition, the cut-off value for each stage with sensitivity ≥90% or specificity ≥90% was added to Additional file 1- Table 1.

**Table 2:** Diagnostic accuracy of the LSM value in detecting each degree of fibrosis

Fibrosis Stage	Cut-off value† (kPa)	AUROC	95% CI	Se (%)	Sp (%)	PPV (%)	NPV (%)	*P* value
≥1	6.3	0.793	0.695–0.871	71.1	75.0	96.7	20.0	0.0002
≥2	7.6	0.764	0.663–0.846	68.0	68.3	72.3	63.6	< 0.0001
≥3	8.3	0.837	0.744–0.906	80.0	76.1	48.5	93.1	< 0.0001
≥4	13.8	0.902	0.822–0.955	80.0	94.2	44.4	98.8	< 0.0001

**†**Cut-off value was optimized by the maximal sum of sensitivity and specificity

* Abbreviations: *AUROC* area under the receiver operator characteristic curve, *95% CI* 95% confidence interval, *Se* sensitivity, *Sp* specificity, *PPV* positive predictive value, *NPV* negative predictive value

### Influence of metabolic indicators on FibroScan in detecting liver fibrosis

**Fig. 2:**
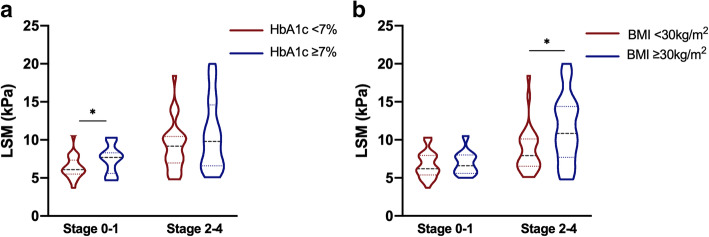
Subgroup analysis grouped by metabolic indicators for liver stiffness measurement (LSM) values in detecting liver fibrosis. Distribution of LSM values subgrouped by (**a**) haemoglobin A1c (HbA1c) < 7% (*n* = 52) or HbA1c ≥7% (*n* = 38) and (**b**) body mass index (BMI) < 30 kg/m^2^ (*n* = 58) or BMI ≥30 kg/m^2^ (*n* = 33). The abscissa represents the presence of significant fibrosis (stage 0–1 vs 2–4), and the ordinate represents the LSM value. Violin plots are shown with median, interquartile range, max and min values. The Mann-Whitney test was used for univariate comparisons between the subgroups. * represents *P* < 0.05

Univariate linear regression between LSM value and metabolic indicators was conducted, with results detailed in Table 3. Variables with *P* < 0.2 were all included in the multivariate backward stepwise linear regression analysis. Variables including age, body mass index (BMI), hypertension, fasting glucose, 2 h glucose, haemoglobin A1c (HbA1c), alanine aminotransferase (ALT) and aspartate aminotransferase (AST), were taken as independent variables, and the LSM value was taken as the dependent variable after adjustment for the histopathological degree of liver fibrosis, ballooning and inflammation, as shown in Table 3. The results demonstrated that HbA1c (β = 0.205, *P* = 0.026) and ALT (β = 0.192, *P* = 0.047) were independently correlated with the LSM value after adjustment for the liver histopathological degree. BMI (β = 0.160, *P* = 0.092) also entered the regression model, while no statistical significance was obtained. To further investigate the influence of metabolic indicators on FibroScan measurements, subgroup analysis of LSM values was conducted grouped by HbA1c < 7% or HbA1c ≥7% and BMI < 30 kg/m^2^ or BMI ≥30 kg/m^2^. The fibrosis stage was divided into stage 0–1 and stage 2–4 (significant fibrosis). The violin plots in Fig. 2a demonstrated a trend that LSM values were higher for patients with HbA1c ≥7% than for patients with HbA1c < 7%, and statistical significance was detected in patients without significant fibrosis. Given that poorly controlled glucose aggravates liver inflammatory activity and inflammation may influence FibroScan measurements, the LSM values between subgroups were matched for histological inflammation and ballooning degree. Interestingly, the increasing trend and statistical significance remained when matching for inflammation or ballooning grade, as shown in Additional file 2- Fig. 1a and b. Subgroup analysis of BMI revealed a similar trend, and statistical significance was detected in patients with significant fibrosis, as shown in Fig. 2b. In view of the results above, an LSM cut-off value in detecting liver fibrosis stage ≥2 grouped by metabolic indicators (HbA1c or BMI) with consistent sensitivity or specificity was then conducted. When the sensitivity or specificity was consistent between subgroups, the cut-off value of significant fibrosis (stage ≥2) for patients with HbA1c ≥7% or BMI ≥30 kg/m^2^ was higher than those with HbA1c < 7% or BMI < 30 kg/m^2^, as shown in Table 4.

**Table 3:** Univariate and Multivariate linear regression analysis of metabolic indicators associated with LSM value

Variable	Univariate linear regression		Multivariate linear regression†	
	Standardized coefficients (β)	*P* value	Standardized coefficients (β)	*P* value
Age	0.231	0.027	–	–
BMI	0.222	0.034	0.160	0.092
hypertension	0.211	0.045	–	–
Fasting glucose	0.222	0.034	–	–
2 h glucose	0.168	0.128	–	–
HbA1c	0.143	0.182	0.205	0.026
Triglycerides	0.023	0.834	–	–
Total cholesterol	0.010	0.928	–	–
LDL cholesterol	0.031	0.780	–	–
HDL cholesterol	0.035	0.748	–	–
ALT	0.150	0.158	0.192	0.047
AST	0.229	0.030	–	–
Fibrosis	0.617	< 0.001	0.502	< 0.001
Inflammation	0.276	0.009	–	–
Ballooning	0.288	0.006	–	–

†The variables with *P* < 0.2 in the univariate regression were included in the multivariate linear regression. The LSM value served as the dependent variable, and age, BMI, hypertension, fasting glucose, 2 h glucose, HbA1c, AST and ALT served as the independent variables after adjustment for liver fibrosis, ballooning and inflammation

* Abbreviations: *BMI* body mass index, *HbA1c* haemoglobin A1c, *ALT* alanine aminotransferase, *AST* aspartate aminotransferase

**Table 4:** LSM cut-off value in detecting liver fibrosis stage ≥2 grouped by metabolic indicators with consistent sensitivity/specificity

Subgroup	Cut-off value† (kPa)	Se (%)	Sp (%)	PPV (%)	NPV (%)
BMI < 30 kg/m^2^	6.2	81.5	50.0	59.5	75.0
BMI ≥30 kg/m^2^	7.5	81.8	63.6	81.8	63.6
HbA1c < 7%	7.7	61.5	80.00	76.2	66.7
HbA1c ≥7%	8.3	60.9	80.00	82.4	57.1

†Cut-off value was determined when the sensitivity or specificity were consistent between subgroups

* Abbreviations: *95% CI* 95% confidence interval, *Se* sensitivity, *Sp* specificity, *PPV* positive predictive value, *NPV* negative predictive value

### Correlation analysis of LSM value with other histologic parameters

Spearman correlation analysis was performed to investigate the relationship between the LSM value of FibroScan and other pathologic degrees, as shown in Additional file 1- Table 2. The LSM value was correlated with the grade of ballooning (r = 0.258, *P* = 0.007) and inflammation (r = 0.241, *P* = 0.013). In addition, the distribution of LSM values classified in accordance with other histological parameters is shown in Additional file 2- Fig. 2. There was a stepwise increase trend as liver ballooning was aggravated, indicating that the LSM value can be used to roughly assess the severity of ballooning, but there was no significant trend in the LSM value with the increase in inflammation or steatosis degree.

## Discussion

This is the first study that evaluated the diagnostic accuracy of FibroScan and obtained the cut-off value for NAFLD patients accompanied by abnormal glucose metabolism. Furthermore, the influence of metabolic indicators on FibroScan measurements was investigated. This cross-sectional study demonstrated that LSM values obtained using FibroScan achieved good diagnostic performance of liver fibrosis, especially in the more severe histologic stage, as the AUROC with a diagnosis of advanced fibrosis (stage ≥3) reached more than 80% and even 90% for cirrhosis (stage ≥4). It is worth noting that the NPV of stage ≥3 was more than 90%, indicating that FibroScan has high efficacy for excluding advanced fibrosis and cirrhosis. In addition, this study suggests that HbA1c was independently associated with the LSM value. The glucose metabolism state may affect LSM value measurement, and its value was elevated in patients with poor glycaemic control, which further emphasized the significance of this study.

Several previous studies have investigated the accuracy of FibroScan in patients with NAFLD, but this study targeted people with NAFLD accompanied by abnormal glucose metabolism [18, 22, 23]. The LSM cut-off values in the study were within the interval of most previous studies, but the value of 8.3 kPa for diagnosing advanced fibrosis was lower than the 9.9 kPa recommended by the *2018 NAFLD guidance*, which may be due to the differences in ethnicity and metabolic state [16]. All subjects in this study were Chinese, had a lower BMI and thinner subcutaneous fat thickness than Americans, and all had abnormal glucose metabolism. Consistent with previous studies, the results further confirmed that FibroScan is more suitable for assessing severe fibrosis, especially for ruling out advanced fibrosis, which can reduce the demand for liver biopsy to some extent.

It has been reported that several factors may affect FibroScan measurements, such as ALT, increased bilirubin levels, excessive alcohol intake and eating. Different cut-off values were suggested in patients with chronic liver disease caused by different aetiologies [24]. However, the effect of metabolic indicators on FibroScan has not been noticed thus far. Based on previous studies, there has been a hypothesis that metabolic indicators may affect the LSM value. As expected, after adjusting the liver pathological degree, HbA1c was significantly associated with the LSM value by multivariate linear regression analysis. There was a significant trend that the LSM value was higher in patients with HbA1c ≥7% than in those with HbA1c < 7%. Although several studies have mentioned the effect of inflammation on FibroScan, and poorly controlled glucose metabolism may lead to more severe liver inflammatory activity, the trend and statistical significance still existed after matching the pathological inflammation and ballooning to counteract their influences. These results implied that inflammation was not the main factor contributing to the effect of glucose metabolism on LSM in this study. However, the potential mechanisms involved remain unknown and need to be further explored. In addition, the LSM cut-off value for significant fibrosis among patients with HbA1c ≥7% was higher than those with HbA1c < 7% in the case of consistent specificity, which meant that different cut-off values should be adopted among patients with different glucose metabolism states. In addition, the ALT level, a typical marker of liver inflammatory activity, was also an independent factor associated with the LSM value in multivariate linear regression analysis in this study. Factors such as hepatocyte swelling, inflammation, and oedema may increase the hydrostatic pressure of the liver in a short time, which results in an increased LSM value [31]. BMI and obesity were reported as independent risk factors for unreliable measurements as well as measuring failure [32]. A prospective multicentre study pointed out that a skin capsular distance (SCD) ≥25 mm led to overestimation of the LSM value [33]. In this study, BMI also affected the LSM measurement, no statistical significance may be due to the small sample size. The cut-off value among patients with BMI ≥30 kg/m^2^ was higher than that among patients with BMI < 30 kg/m^2^ when the sensitivity between these two subgroups were consistent. The effect of BMI may be attributed to its influence on both subcutaneous fat thickness and metabolic state. In general, the study provided a new perspective that glucose metabolism may influence the LSM value obtained from FibroScan. The current optimal cut-off values may be unsuitable for patients with abnormal glucose metabolism. Different cut-off values need to be considered for different glucose metabolic states in clinical applications. This is also the reason specific cut-off values are given for this study population. Clinically, stratified assessment of LSM based on glucose metabolic state is more practical. However, the results need to be further confirmed by expanding the subject number in the future.

### Study strengths and limitations

The strengths of this study were that the degree of fibrosis was obtained from liver biopsy, a gold standard, and it is the first study to propose the effect of glucose metabolism on the LSM value and prove it to a certain extent. However, there were several limitations in the study. First, this study was cross-sectional, which can only describe the phenomenon. The effect of glucose metabolism on LSM value needs to be further examined in a longitudinal study. Second, the sample size was small and the data featured skewed distribution. Most of the subjects were patients with moderate NASH, with a relative lack of NAFL and severe fibrosis, as liver biopsy is not recommended for these parts of the population according to guidelines. Therefore, the study revealed a general trend, but subgroup analysis cannot be performed in each fibrosis stage. More detailed subgrouping and cut-off value comparison for each degree should be carried out by expanding the number of subjects. The last limitation was the lack of the use of XL probe from FibroScan. The XL probe, which is recommended for severe obese patients, can increase the rate of reliable results among them, but it is unfortunate that XL probe had not been equipped during the period of this study. Most of the patients in this study were mildly obese rather than morbidly obese. Given that previous studies with similar BMI adopted M probe, the results obtained by M probe in this study are also reliable [18, 23]. However, the use of XL probe for obese patients will be more suggested if conditions are available in the future.

## Conclusions

In conclusion, FibroScan was confirmed to be a relatively accurate diagnostic approach for evaluating liver fibrosis among NAFLD patients with abnormal glucose metabolism. It is valuable for evaluating severe fibrosis, especially for excluding advanced fibrosis. The glucose metabolic state may affect the LSM value, and the LSM values are higher in patients with HbA1c ≥7%. This study provides clinical advice for the application of FibroScan in the diagnosis and evaluation of NAFLD patients, especially in patients with abnormal glucose metabolism. It is worth noting that the glucose metabolism state should be considered in the clinical application of FibroScan.

## Supplementary Information

**Additional file 1: Table 1.** Diagnostic Accuracy of LSM in Detecting Each Degree of Liver Fibrosis with Sensitivity or Specificity ≥90%. Table 2. Correlation analysis of LSM value with other histopathologies of NAFLD.

**Additional file 2: Fig. 1.** Subgroup analysis grouped by Hemoglobin A1c (HbA1c) for liver stiffness measurement (LSM) values in detecting liver fibrosis after matching for (a) inflammation and (b) ballooning. Distribution of LSM values subgrouped by (a) haemoglobin A1c (HbA1c) <7% (n=13vs13) or HbA1c ≥7% (n=16vs16) and (b) HbA1c <7% (n=14vs14) or HbA1c ≥7% (n=22vs22), respectively. The abscissa represents whether the presence of significant fibrosis (stage 0-1 vs 2-4) and the ordinate represents LSM value. Violin plots were showed with median, interquartile range, max and min values. Mann-Whitney test was used for univariate comparison between subgroups. * represents p <0.05. **Fig. 2.** The distribution of liver stiffness measurement (LSM) value differentiated in accordance to other histological parameters. The abscissa represents the liver (a) ballooning, (b) inflammation and (c) steatosis grade, and the ordinate represents LSM value. Boxplots were showed with median, interquartile range, 5 and 95% percentile. ▲ represents the value of greater variability. Kruskal-Wallis test with Dunn’s multiple correction were used for univariate comparisons between groups. * represents p <0.05.

## Data Availability

The datasets generated and/or analysed during the current study are available from the corresponding author on reasonable request.

## Abbreviations

NAFLD: Nonalcoholic fatty liver disease
HS: Hepatic steatosis
NAFL: Nonalcoholic fatty liver
NASH: Nonalcoholic steatohepatitis
DM: Diabetes mellitus
T2DM: Type 2 diabetes mellitus
LSM: Liver stiffness measurement
ALT: Alanine aminotransferase
NAS: The NAFLD activity score
IQR: Interquartile range
ROC curve: Receiver operating characteristic curve
AUROC: Area under the ROC curve
PPV: Positive predictive value
NPV: Negative predictive value
BMI: Body mass index
HbA1c: Haemoglobin A1c
TG: Triglyceride
TC: Total cholesterol
AST: Aspartate aminotransferase
