# Supplementary material for: Performance of liver stiffness measurements obtained with FibroScan is affected by glucose metabolism in patients with nonalcoholic fatty liver disease
Source: Lipids Health Dis. 2021 Mar 23;20:27. doi: 10.1186/s12944-021-01453-5 (PMC7986416; doi:10.1186/s12944-021-01453-5)
Supplement: Supplementary file 1 — Additional file 1: Table 1. Diagnostic Accuracy of LSM in Detecting Each Degree of Liver Fibrosis with Sensitivity or Specificity ≥90%. Table 2. Correlation analysis of LSM value with other histopathologies of NAFLD. [file 12944_2021_1453_MOESM1_ESM.docx]

**Table 1.** Diagnostic Accuracy of LSM in Detecting Each Degree of Liver Fibrosis with Sensitivity or Specificity ≥ 90%

| Fibrosis Stage | Sensitivity ≥ 90% | | | | |  | Specificity ≥ 90% | | | | |
| --- | --- | --- | --- | --- | --- | --- | --- | --- | --- | --- | --- |
|  | Cut-off value (kPa) | Se (%) | Sp (%) | PPV (%) | NPV (%) |  | Cut-off value (kPa) | Se (%) | Sp (%) | PPV (%) | NPV (%) |
| ≥1 | 4.8 | 96.4 | 37.5 | 94.1 | 50.0 |  | 8.1 | 44.6 | 100.0 | 100.0 | 14.8 |
| ≥2 | 5.9 | 90.0 | 36.6 | 63.4 | 75.0 |  | 8.3 | 58.0 | 90.2 | 87.9 | 63.8 |
| ≥3 | 7.6 | 90.0 | 59.2 | 38.3 | 95.5 |  | 10.4 | 60.0 | 91.6 | 66.7 | 89.0 |
| ≥4 | 8.3 | 100.0 | 67.4 | 15.2 | 100.0 |  | 13.8 | 80.0 | 94.2 | 44.4 | 98.8 |

AUROC, area under the receiver operator characteristic curve; 95% CI, 95% confidence interval; Se, sensitivity; Sp, specificity; PPV, positive predictive value; NPV, negative predictive value.

**Table 2.** Correlation analysis of LSM value with other histopathology of NAFLD

| Histopathology | r | *P* value |
| --- | --- | --- |
| Ballooning | 0.258 | 0.007 |
| Inflammation | 0.241 | 0.013 |
| Steatosis | 0.145 | 0.139 |

Bivariate Spearman correlation of LSM value and other histologic parameters, respectively.

r, correlation coefficient.
