# Supplementary material for: Performance of liver stiffness measurements obtained with FibroScan is affected by glucose metabolism in patients with nonalcoholic fatty liver disease
Source: Lipids Health Dis. 2021 Mar 23;20:27. doi: 10.1186/s12944-021-01453-5 (PMC7986416; doi:10.1186/s12944-021-01453-5)
Supplement: Supplementary file 2 — Additional file 2: Fig. 1. Subgroup analysis grouped by Hemoglobin A1c (HbA1c) for liver stiffness measurement (LSM) values in detecting liver fibrosis after matching for (a) inflammation and (b) ballooning. Distribution of LSM values subgrouped by (a) haemoglobin A1c (HbA1c) <7% (n=13vs13) or HbA1c ≥7% (n=16vs16) and (b) HbA1c <7% (n=14vs14) or HbA1c ≥7% (n=22vs22), respectively. The abscissa represents whether the presence of significant fibrosis (stage 0-1 vs 2-4) and the ordinate represents LSM value. Violin plots were showed with median, interquartile range, max and min values. Mann-Whitney test was used for univariate comparison between subgroups. * represents p <0.05. Fig. 2. The distribution of liver stiffness measurement (LSM) value differentiated in accordance to other histological parameters. The abscissa represents the liver (a) ballooning, (b) inflammation and (c) steatosis grade, and the ordinate represents LSM value. Boxplots were showed with median, interquartile range, 5 and 95% percentile. ▲ represents the value of greater variability. Kruskal-Wallis test with Dunn’s multiple correction were used for univariate comparisons between groups. * represents p <0.05. [file 12944_2021_1453_MOESM2_ESM.docx]

**
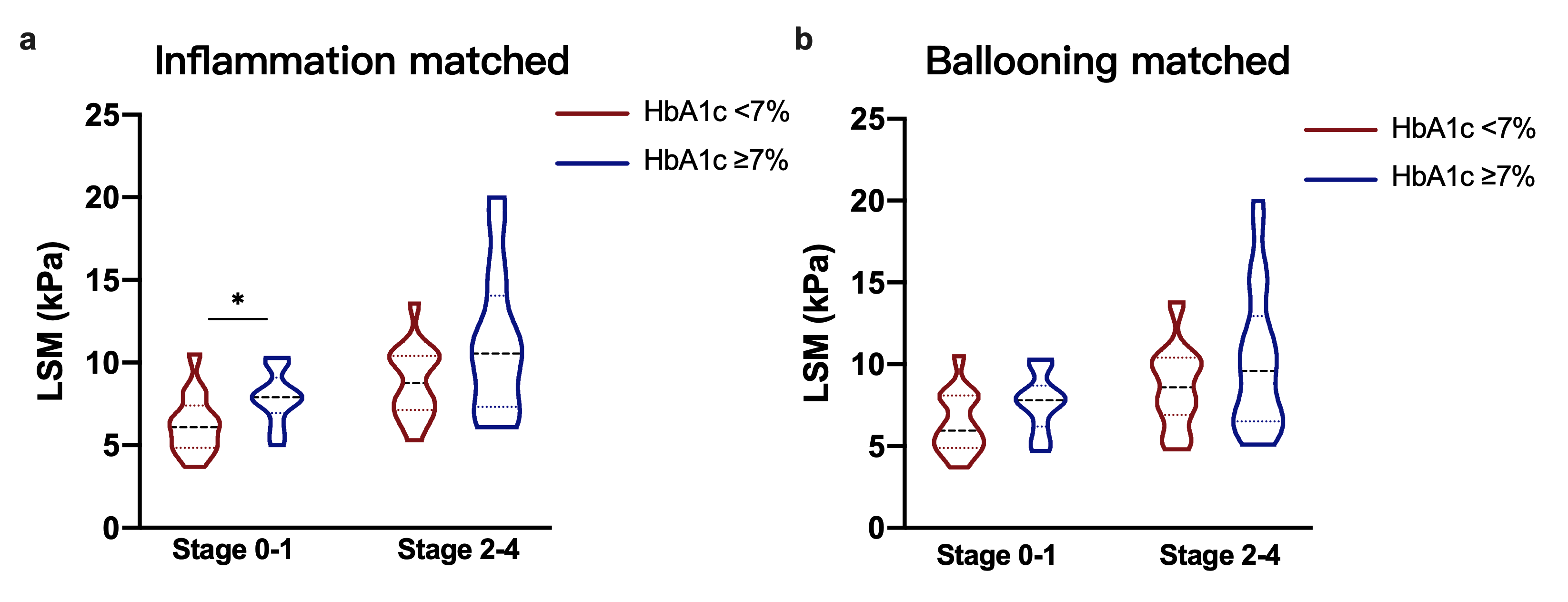
**

**Fig. 1.** Subgroup analysis grouped by Hemoglobin A1c (HbA1c) for liver stiffness measurement (LSM) values in detecting liver fibrosis after matching for (a) inflammation and (b) ballooning. Distribution of LSM values subgrouped by (a) haemoglobin A1c (HbA1c) <7% (n=13vs13) or HbA1c ≥7% (n=16vs16) and (b) HbA1c <7% (n=14vs14) or HbA1c ≥7% (n=22vs22), respectively. The abscissa represents whether the presence of significant fibrosis (stage 0-1 vs 2-4) and the ordinate represents LSM value. Violin plots were showed with median, interquartile range, max and min values. Mann-Whitney test was used for univariate comparison between subgroups. * represents p <0.05.

**
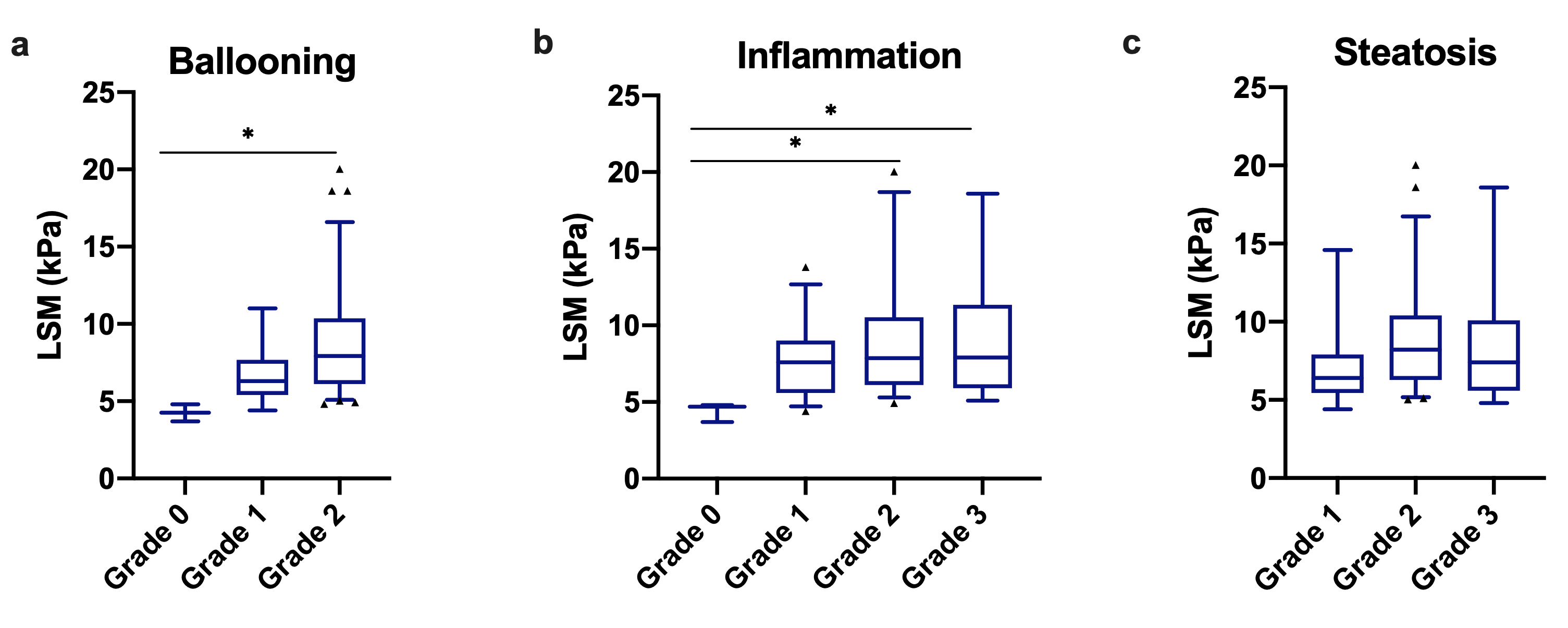
**

**Fig. 2.** The distribution of liver stiffness measurement (LSM) value differentiated in accordance to other histological parameters. The abscissa represents the liver (a) ballooning, (b) inflammation and (c) steatosis grade, and the ordinate represents LSM value. Boxplots were showed with median, interquartile range, 5 and 95% percentile. ▲ represents the value of greater variability. Kruskal-Wallis test with Dunn’s multiple correction were used for univariate comparisons between groups. * represents *p* <0.05.
